# Structural insights into the HNF4 biology

**DOI:** 10.3389/fendo.2023.1197063

**Published:** 2023-06-19

**Authors:** Brice Beinsteiner, Isabelle M. L. Billas, Dino Moras

**Affiliations:** ^1^ Laboratory IGBMC (Institute of Genetics and of Molecular and Cellular Biology), Centre for Integrative Biology (CBI), Illkirch, France; ^2^ Université de Strasbourg (Unistra), Strasbourg, France; ^3^ Institut National de la Santé et de la Recherche Médicale (INSERM) U1258, Illkirch, France; ^4^ Centre National de la Recherche Scientifique (CNRS) UMR 7104, Illkirch, France

**Keywords:** hepatocyte nuclear factor 4, HNF4, MODY1, nuclear receptors, transcription, protein-DNA complexes, DNA Allostery, structural biology

## Abstract

Hepatocyte Nuclear Factor 4 (HNF4) is a transcription factor (TF) belonging to the nuclear receptor (NR) family that is expressed in liver, kidney, intestine and pancreas. It is a master regulator of liver-specific gene expression, in particular those genes involved in lipid transport and glucose metabolism and is crucial for the cellular differentiation during development. Dysregulation of HNF4 is linked to human diseases, such as type I diabetes (MODY1) and hemophilia. Here, we review the structures of the isolated HNF4 DNA binding domain (DBD) and ligand binding domain (LBD) and that of the multidomain receptor and compare them with the structures of other NRs. We will further discuss the biology of the HNF4α receptors from a structural perspective, in particular the effect of pathological mutations and of functionally critical post-translational modifications on the structure-function of the receptor.

## Introduction

Hepatocyte nuclear factor 4 (HNF4) belongs to the large superfamily of nuclear receptors (NRs) that regulate diverse biological activities from embryonic development, growth, reproduction and adult physiology (1, 2). HNF4 is encoded by two genes, *HNF4α* and *HNF4γ*, giving rise to two protein subtypes, HNF4α (NR2A1) and HNF4γ (NR2A2) (3). HNF4 is abundantly expressed in the liver, pancreas, kidney, stomach, small intestine and colon (4–6) and is essential for development and the homozygous loss of functional HNF4 protein causes embryonic lethality of the mutant mice (7). HNF4 is also essential for the normal function of hepatocytes and pancreatic β-cells by regulating numerous crucial genes involved in glucose, cholesterol, and fatty acid metabolism (8). Whereas mutations found in the HNF4γ gene have not yet been firmly linked to human diseases, dysregulation of HNF4α is undoubtedly associated to some important pathologies. In particular, mutations of the HNF4α gene are found in human populations carrying the syndrome of maturity onset diabetes of the young 1 (MODY1), while mutations in the HNF4α-binding sites located in the promoter/enhancers of genes coding for factors VII and IX are linked to hemophilia (9). In addition, HNF4α is increasingly being linked to liver and colon cancer (10).

Like most other members of the NR superfamily, HNF4^1^ is characterized by the presence of two highly conserved functional and structural domains, a DNA binding domain (DBD) and a ligand binding domain (LBD) at the C-terminal end of the protein. In addition, it is interesting to note that HNF4 is characterized by the presence of a long C-terminal F domain of about 80-100 residues that is predicted to be essentially disordered, except in the case of highly derived insects, such as *Drosophila melanogaster*, where an insertion of 100-200 residues is found that AlphaFold (11) predicts to be a helical region. The F domain is important for the function of HNF4, being the site of interactions with various transcriptional regulators - see the BioGRID database (https://thebiogrid.org)- including for example the Pancreatic Islet LIM HD Transcription Factor Isl1 (12). Of note, HNF4 can functionally interact with both coactivators and corepressors without altering the status of any putative ligand and the presence of the F domain may play a role in discriminating between the different coregulators (13).

**Figure 1:**
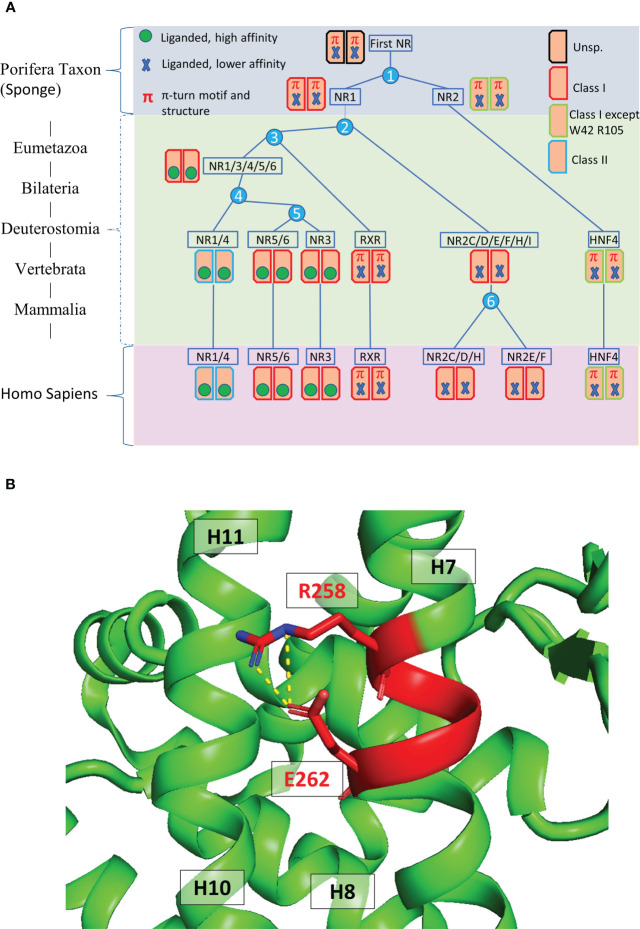
**(A)** Evolutionary history of the major human receptor families with respect to the existence of the π-turn motif in the protein sequence as well as in the protein structure and the corresponding dimerization properties. The NR LBD is illustrated by a rectangle, whose color corresponds to the class marker properties: red (e.g. RXR) when all class I markers are present, green (e.g. HNF4) when class I markers are present, except W109/40 and R321/105, and blue (e.g. NR1/4) when class II markers are present. Numbers in the blue circles correspond to the number of duplication events. The green circle inside the rectangle indicates the presence of high-affinity, specific ligand and the blue cross indicates the presence of less specialized, lower affinity ligand inside the ligand-binding pocket. **(B)** 3D representation of the π-turn (in red) in the H7 helix of HNF4 (PDB 4IQR). The residues Arg258 (Arg267) and Glu262 (Glu271) form a salt bridge.

NRs are found in all metazoan organisms with a number that ranges from about 20 in insects to about 48 to 70 in vertebrates (14–16). Sponges that are thought to be the earliest metazoan phyla (Porifera) contain two genes coding for the most basal NRs called SpNR1 and SpNR2. These two NRs are reminiscent of RXR for SpNR1 and HNF4 for SpNR2 and define the two major subdivisions in the NR evolutionary tree, one for SpNR2/HNF4 (subfamily NR2A) and the other one for SpNR1/RXR (subfamily NR2B) and all other NRs (see **Figure 1A**). HNF4 is thus a basal NR whose sequence has remained extremely well conserved in the more than 600 million years that separate humans from sponges (17–19). A recent integrated phylogenetic, sequence and structural analysis revealed that the two most basal NRs, RXR and HNF4, and only them, contain a peculiar structural feature in the helix 7 of the LBD characterized by a conserved RxxxE motif with an associated π-helical geometry (20). This study highlighted the importance of this structural π-helical feature for the stabilization of the LBD and the formation of a stable interface in the LBD homodimer, the functional and transcriptionally active form of the HNF4 receptor (**Figure 1B**). This review will discuss the biology of the HNF4α receptors from a structural perspective, in particular the effect of pathological mutations on the structure-function of the receptor. Here, we will discuss the isolated HNF4 DBD and LBD domains and the multi-domain receptor and highlight the structural consequences that disease mutations found in HNF4 have on the receptor function.

### HNF4 ligand binding domain

**Figure 2:**
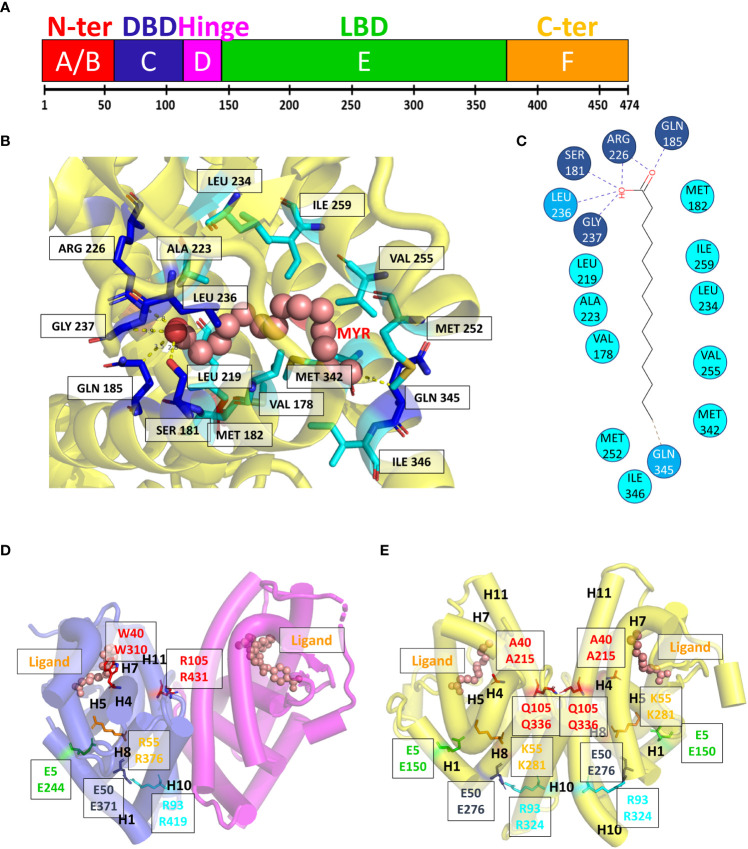
Domain structure and ligand binding pocket. **(A)** One-dimensional representation of the human HNF4α. The DNA binding domain (DBD, or C-domain) is shown in dark blue. The hinge region (D-domain) is shown in magenta. The ligand binding domain (LBD or E-domain) is shown in green. The A/B domain at the N-terminal end is shown in red and the F domain at the very C-terminal end is shown in orange. **(B, C)** Two- and three-dimensional representations of the ligand environment inside the HNF4α pocket (PDB: 4IQR). The myristic acid ligand is shown in a sphere representation. Amino acid residues that form H-bonds with the ligand are shown in dark blue, the Van des Waals contacts with the ligand are shown in blue. Amino acid residues that form hydrophobic interactions with the ligand are shown in cyan. **(D, E)** Class I markers on **(D)** the RXR-RAR LBD heterodimer (PDB code 1DKF) and **(E)** the HNF4α-HNF4α LBD homodimer (PDB code 4IQR). For all class markers two different ways of numbering residues are used. The first one indicates the residue label according to the original paper, the second one refers to the residue number in the respective PDB file. The labels of class markers mutants Trp40 to Ala in H5 and Arg105 to Gln in H11 are shown in red. The mutation Trp40 to Ala created void volume inside the ligand binding pocket. The residue Arg105 is involved in the dimerization interface and its mutation to Gln is likely to weaken the dimerization strength. The ligands shown with a sphere representation, are shown inside the ligand binding pocket.

At the C-terminal end of NRs is the LBD which is crucial for ligand binding, receptor dimerization and interaction with transcriptional coregulators (**Figure 2A**) (21–24). The structures of the LBD of HNF4α and HNF4γ, resolved by X-ray macromolecular crystallography, are very similar and show the presence of co-crystallized fatty acids originating from the bacterial expression host (**Figures 2B, C**) that are strongly bound in the ligand binding pocket and hard to displace (24, 25). In that respect, they act more like entities stabilizing the protein conformation rather than classical ligands that trigger the receptor transcriptional activities. On the other hand, another study reported that in a mammalian cell culture, as well as in the mouse liver, HNF4α binds in a reversible fashion the essential fatty acid linoleic acid (LA, C18:2) that thus appears to be the endogenous HNF4 ligand (26). Importantly, this suggests that molecules could be found that would potentially modulate the receptor transcriptional activity or its level of expression and thus that HNF4 could be a potential target for pharmacological compounds, as later was shown to be the case (10, 27).

The analysis of the LBD sequences based on differentially conserved residues (i.e. residues strictly conserved in one class while strictly absent from the other one) leads to a partition of the family into two classes (20, 28–30). Class I NRs, such as HNF4 and RXR, behave either as monomers or homodimers, while class II receptors encompass all NRs that heterodimerize with RXR. Class I and class II NRs exhibit a set of conserved class-specific residues that form distinct communication pathways. In class I NRs, the latter links together helix 1 to the dimerization interface *via* helix 8, notably *via* Glu5, Trp40, Lys/Arg55, Arg/Lys93, Arg105 for class I [according to the nomenclature in (28)].Note that SpNR1/RXR encompasses all class I markers, while in SpNR2/HNF4, Trp40 and Arg105 are missing (**Figures 2D, E**). These specific features could then explain the absence of HNF4 heterodimers. First, the residue Arg105 in H10, that is crucial for dimerization, is replaced by a glutamine residue (Gln345) which is then associated with Glu42 (Glu226)2, thus affecting the dimerization properties observed for other NRs. In addition, numerous H-bonds linking H9 and H10 of the LBD partners, mostly absent in RXR, are largely responsible for the strength of HNF4 homodimerization. Finally, the replacement of Trp40 by an alanine residue would disrupt the allosteric control and affect the π-turn specific signaling by heterodimers. In fact, in RXR, Trp40 contacts the envelope of the ligand pocket and was shown to be part of an important allosteric network between heterodimeric partners, ligands and coregulator-binding sites (31). Mutations of these two highly conserved residues Trp40 and Arg105 in HNF4a highlight the early divergence of this receptor from the rest of the family and their functional importance, in particular for dimerization.

A comparative analysis of the LBD crystal structures deposited in the PDB database revealed a peculiar feature, which is the presence of a helical deformation called π-turn or α/π-bulge within the α-helix 7 of RXR-USP and HNF4 (**Figure 1B**). A conserved RxxxE motif where the two invariant residues Arg and Glu form an intra-helical salt bridge further characterizes this specific conformation (20). The π-type helical structures being thermodynamically less stable than α-helices they are favored only when they are associated with a functional advantage. The important role of the π-turn in the dimerization process of the LBDs and the related stability of the HNF4 dimers suggests it is most probably the functional correlation (20). Residues from the π-turn and the helices H9 and H10 of both subunits link together the two LBD subunits. In HNF4 the numerous contacts result in a larger buried surface at the dimer interface consistent with an energetically more stable oligomer. The functional importance of the HNF4 π-turn to the homodimerization process was demonstrated in earlier studies, where the π-turn residues Arg202 (Arg267) and Glu206 (Glu271) were mutated and the functional consequence assessed (32). In this work, it was shown that removing the charges of Arg202 (Arg267) and Glu206 (Glu271) affects dimerization of the protein in solution and affect the HNF4α transcriptional activity in a variety of different cell lines. The impairment on transcriptional activity is even larger with the deletion mutant ΔGlu206 (Glu271 in hHNF4α), which was also shown to be less efficient in recruiting transcriptional partners, such as SRC-1 and PGC-1.

### HNF4 DNA binding domain

Homodimerization of the receptor is attributed not only to the LBD dimerization, but also to the specific binding of HNF4 DBD to response elements in the promoter or the enhancer regions of target genes. HNF4 preferentially recognizes binding sites composed of a tandem repeat of two hexanucleotide half-sites of the type A/GGGTCA spaced by one nucleotide (DR1). This type of DR1 response elements is also recognized by RXR and COUP-TF2 homodimers (33–35). Initial *in vitro* protein binding microarrays (PBM) identified an extensive repertoire of HNF4α binding sites and new rules for HNF4α DNA binding (34, 36). The results indicated that HNF4 binds DR1 response elements (AGGTCAxAGGTCA), but with a strict preference at the 3’ side for a binding motif of the type xxxCAAAGTCCA (called HNF4-specific binding motif, H4-SBM). Interestingly, ChIP-seq data analysis supported this view and importantly showed that the H4-SBM is exclusively bound by HNF4, and not by RXR or other NRs (34). More recently, genome-wide studies of murine epithelial tissues, including intestine and kidney, showed similar H4-SBM binding motifs in the accessible chromatin regions of the brush border genes regulated by HNF4 (37), further supporting the *in vivo* relevance of this observation.

**Figure 3:**
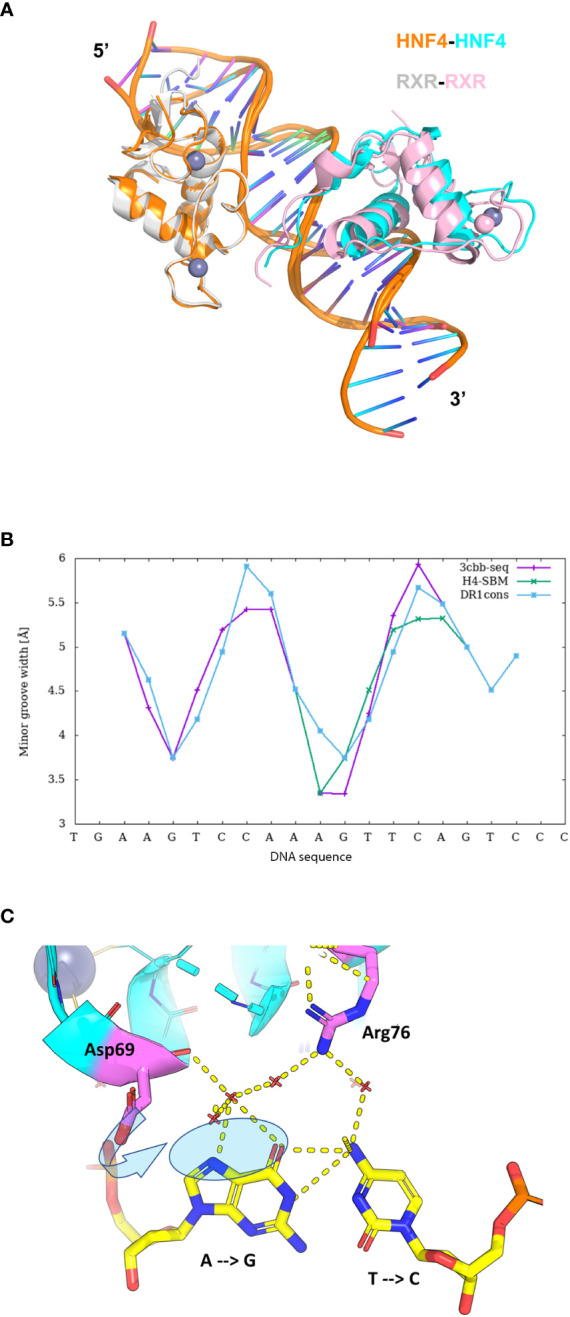
Structural analysis of the HNF4α DBD homodimer on a DR1 RE. **(A)** Superimposition of the homodimer structures of HNF4 DBD (PDB code 3CBB) and RXR DBD (PDB code 6FBQ) bound to their cognate DR1 RE, taking the 5’ subunit as the reference. The two structures adopt the same global topology when bound to their cognate DR1 RE, but show a difference in the positioning of the 3’-DBD subunits. The DBD are represented as ribbon, with the 5’-DBD in orange and white for HNF4 and RXR, respectively and the 3’- DBD in cyan and pink for HNF4 and RXR, respectively. The zinc ions are depicted with spheres with the same color as the respective DBD subunit. The DNA directionality is indicated with 5’ and 3’. **(B)** Sequence dependent DNA Minor Groove Width values (in Å) calculated with DNAshape (41) for the sequence of HNF4 DBD (3cbb-seq) (PDB code 3CBB) in magenta, compared to the HNF4-specific sequence H4-SBM in green and the consensus DR1 sequence (DR1cons) in light blue. **(C)** Model of the potential interactions that the HNF4 specific residues Asp69 (Asp78) and Arg76 (Arg85) would make with a C nucleotide at position +4, as present in the HNF4 specific H4-SBM motif. The model is based on the HNF4 DBD structure where the T is mutated to C. The large negative charge on the major groove of G, shown with a transparent blue ellipse, would drive a different conformation of Asp69 (indicated with an arrow), optimal for interactions with G.

The crystal structure of HNF4α DBD bound to a cognate DR1 response element was solved in 2008 (38). It is the only one available bound to a natural binding sequence found in the human *HNF1a* promoter. In addition, the crystal structure of the HNF4α multidomain receptor, comprising the DBD and LBD domains, was solved bound to a consensus (and thus unnatural) DR1 response element (called DR1cons) (39). It will be discussed in more detail in the next section and compared with the HNF4 DBD homodimer structure. Here we propose to compare the structure of the HNF4 DBD homodimer to the one adopted by the homodimer of RXR DBD bound to its cognate DR1 RE (PDB code 6FBQ) (40). These two different DBD structures, both bound to a DR1 RE adopts the same global topology. However, the superimposition of the two DBD homodimer structures, taking the 5’ subunit as the reference, shows a difference in the positioning of the 3’-DBD subunit (**Figure 3A**).

The HNF4α recognition DNA sequence element used for the crystallization of HNF4α DBD homodimer, called 3cbb-seq, reads 5’-AG*TC*CAAAG*T*TCA-3’. Thus, it differs from DR1cons at positions -4, -3, +3 (shown in italics), with position 0 being the spacer nucleotide. Moreover, it also differs from the H4-SBM motif (xxxCAAAGTCCA) at position +4 where C is replaced by T. Since the DNA acts as an allosteric effector for protein binding, changes in its mere sequence, impact the parameter values characterizing the DNA geometrical shape (42), such as Minor Groove Width (MGW), Roll, Propeller Twist (ProT) and Helix Twist (HelT). These parameters are usually calculated at each bp position as a function of the pentameric environment (41). We illustrate this in the case of the DNA sequence chosen for the DBD structure (3cbb-seq) compared to the HNF4-specific sequence H4-SBM and the DR1cons, as shown in **Figure 3B**. We observe a sequence-dependent behavior for all shape parameters, in particular for the MGW which has a strong and sharp minimum at position +1 for the H4-SBM sequence, and overall smaller MGW values compared to those of the 3cbb-seq and DR1cons sequences. This is an important difference, because minima in the MGW correlate with regions of enhanced negative electrostatic potential and represent binding sites for the side chains of basic amino acid residues, such as lysine and arginine residues. This suggests that the HNF4-specific sequence is tailored for the specific binding of its receptor.

The structure of HNF4 DBD on the 3cbb-seq reveals an asymmetric homodimer bound to the DR1 response element, where each monomer adopts a tertiary structure composed of a N-terminal β-hairpin, two α-helices perpendicular to each other followed by a 3_10_-helix turn and the C-terminal extension that encompasses the T-box region (38). The HNF4 DBD homodimerization interface is made by the C-terminal T-box of the 3’-DBD subunit and the second Zn finger (Zn-II) region of the 5’-DBD. This contrasts with the organization of homodimeric steroid receptor DBD, such as ER or GR (43, 44), where the dimerization interface is symmetrically formed by the D-box region of each of the two homodimer subunits and covers the majority of the minor groove of the spacing base pair. The HNF4 DBD homodimer on DR1 RE is thus asymmetric in nature and the protein-protein interactions formed in the presence of DNA provide a high degree of cooperativity.

The binding specificity of HNF4 for H4-SBM compared to the 3cbb-seq and the DR1cons sequences was attributed to two residues in the recognition helix H1, Asp69 (Asp78) and Arg76 (Arg85) that are unique to HNF4 and replaced by Glu and Lys, respectively, in RXR or COUP-TF (34). Unfortunately, no structure of HNF4 DBD exists in complex with the H4-SBM motif. However, we can analyze the potential impact of replacing T at position +4 in 3cbb-seq by C, as found in the preferred H4-SBM sequence when virtually mutating the corresponding nucleotides in the crystal structure of HNF4 DBD. One can observe that the Asp69 (Asp78) and Arg76 (Arg85) are located at the level of the nucleotide at position +4, Arg76 (Arg85) closer to C and Asp69 (Asp78) closer to its complementary G nucleotide (**Figure 3C**). The Asp69 (Asp78) residue would interact in an optimal manner with the large negative charge on the major groove of G, much better than with the smaller negative and positive charges scattered over the surface of the A complementary to the +4 T of the 3cbb-seq DNA. Given the overall DNA shape, it is likely that a Glu residue (as found in RXR, COUP-TF and other NRs) instead of Asp (unique to HNF4) would penetrate too deep inside the major groove to make adequate interactions with G.

### HNF4 multidomain receptor

**Figure 4:**
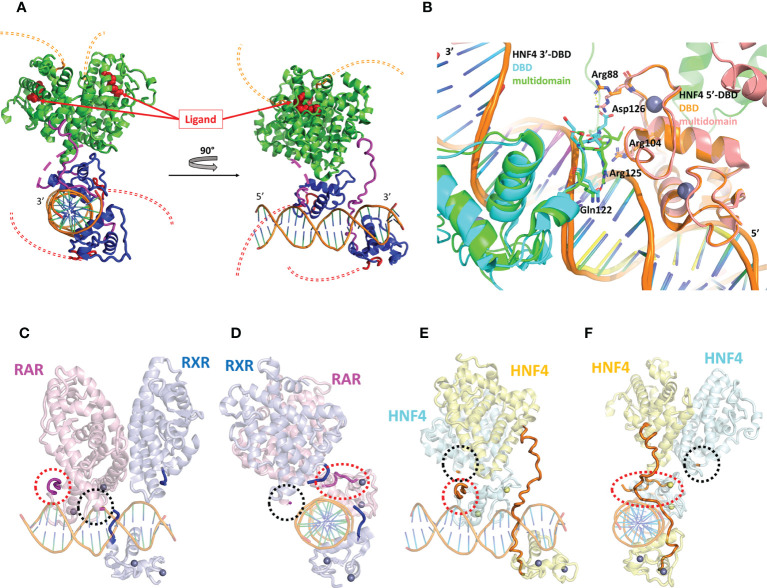
Structural analysis of the multidomain HNF4α homodimer on a DR1 RE. **(A)** Three-dimensional representation of the HNF4α multidomain complex with DR1cons and coactivator peptides (PDB code 4IQR). The DBD is shown in dark blue and the hinge region in magenta. For one of the two monomers the hinge region which is not visible in the electron density (and thus not modelled) is shown with a dotted line. The LBD is shown in green. The A/B domain at the N-terminal end and the F domain at the vey C-terminal end of the receptor are not present in the constructs used for crystallization and their potential location is putatively shown with a double thin dotted red and orange line respectively. The ligand inside the ligand binding pocket of the LBD is displayed as red spheres. **(B)** Zoom on the DBD part of the superimposition of the homodimer structures of the multidomain HNF4α (PDB code 4IQR) and DBD (PDB code 3CBB) bound to DR1 RE, taking the 5’ subunit as the reference. The proteins are represented as a cartoon representation, with the 5’-subunit in orange and pink for HNF4 in the DBD and in the multidomain structures, respectively and the 3’-subunit in cyan and green for HNF4 in the DBD and in the multidomain structures, respectively. The zinc ions are depicted with spheres with the same color as the respective subunit. The DNA directionality is indicated with 5’ and 3’. **(C-F)**. Two different views of **(C, D)** the multidomain RXR-RAR heterodimer in complex with a consensus DR1 RE (PDB: 5UAN) and **(E, F)** the multidomain HNF4α homodimer in complex with a consensus DR1 RE (PDB: 4IQR). The positions of the hinge regions (D domains) are highlighted with thick lines or with dotted circles for hinge regions that were not built, as it is the case for RXR-RAR/DR1 and for one of the HNF4α subunit (not built in the electron density, reflecting disorder and flexibility). The two N- and C-termini of the hinge region of RXR are indicated in dark blue and those of RAR in magenta. For HNF4α, the hinge region that is almost completely built in the electron density is shown in orange. For the other subunit of the HNF4α homodimer, the N- and the C-termini are indicated with a red and with a black dotted ellipse, respectively.

Most structural studies of HNF4 have focused on its individual DBD and LBD structures (24, 25, 38), unveiling the molecular mechanisms of receptor dimerization, ligand binding and DNA recognition. However, the molecular basis for the understanding of the crosstalk between the DBD and the LBD crucial for fine tuning of gene expression can only be gained with the structure-function studies of the full receptor. This was in some part achieved thanks to the resolution of the X-ray structure of the DBD-hinge-LBD regions of the HNF4α receptor bound to a (unnatural) DR1cons element (AGGTCAAAGGTCA) and coactivator peptides (39) as shown in **Figure 4A**. Note that the construct used for the multidomain HNF4 crystal structure does not encompass the N-terminal A/B region (ΔAB), nor the long C-terminal F-region (ΔF). The crystal structure shows the topological arrangement of the two homodimer domains (DBD and LBD) on the DR1cons DNA, with the LBD dimer sitting on the DBD dimer and making extensive interactions through helix H9 and the loop 9-10 of the 3’-LBD with the 5’-DBD. Note however that the hinge regions of the two subunits are not completely visible in the crystal structure, due to poor electron density likely due to disorder or to various conformations co-existing in the different molecules of the crystal. In particular, a long stretch of 17 amino acid residues in the hinge of the 5’-DBD could not be modeled, while the hinge of the 3’-DBD is built, even though the quality of the electron density is poor (with a huge disorder reflected by large B factor values), questioning the relevance of the precise conformation. In fact, the part of the hinge of the 3’-DBD is structured because of interactions, on the one hand between the N-terminal part of the hinge region and the DNA minor groove and the 5’-DBD and on the other hand between the C-terminal part of the hinge region and one subunit of a nearest neighbor dimer (crystal packing interactions).

It is interesting to note that the hinge of HNF4 is a sensitive region and cannot be thought of as a mere linker between the DBD to the LBD, as seen by the fact that residue mutations and post-translational modifications seriously affect the function of the receptor, as will be discussed below. In fact, the hinge of HNF4 is the shortest of all NRs, with an average size of 17 amino acid residues, compared to a length between 20 and 50 residues in all other NRs (**Table 1**). This short hinge is likely to contribute to the specificity in the binding to the DR1 RE, since it acts as a tensed spring between the 3’-DBD and -LBD domains and it is likely to be ideally positioned when bound to the preferred DR1 H4-SBM sequences. Unfortunately, the structure of the multidomain HNF4 homodimer was solved on the ideal consensus DNA sequence DR1cons which does not represent a biologically relevant DNA binding site for HNF4. This has to be kept in mind, since this could affect the overall topology of the full receptor on DNA and bias the analysis. When superimposing the DBD part of the multidomain HNF4 homodimer (39) with the structure of the DBD homodimer on 3cbb-seq DNA (38), we notice differences in the positioning of the 3’-DBD as shown in **Figure 4B**, likely due to differences in the DNA sequences, as discussed above. As a consequence, for example, the positioning of the key hinge residue Arg125 (Arg134) in the minor groove is different and the interactions of Asp126 (Asp135) with Arg88 (Arg97) of the 5’-DBD, described in the original DBD crystal structure (38), does not take place in the multidomain structure.

**Table 1:** Average size of different nuclear receptor domains.

Nuclear receptor	A/B	C	D	E	F
NR2A1_HNF4α	57	72	17	224	99
NR2B1_RXRα	135	72	21	226	6
NR3C1-4 (Oxosteroid)	420-550	72-75	35-50	231-232	19-20
NR3A1_ERα	182	72	51	235	50
NR3B1_ERRα	76	72	39	226	4
NR1I1_VDR	21	72	28	297	4
NR1B1_RARα	85	72	20	233	47
NR1C1_PPARα	99	71	26	264	3
NR4A2_NURR1	260	72	25	230	6
NR5A1_SF1	10	72	136	234	4

The average is calculated only from the sequence of the main model mammals. For HNF4, there are significant variations in some taxa, such as insects. But overall the values here are representative of the majority of taxa.

To further deepen our understanding of the topology of the full receptor on its cognate binding site, it is interesting to compare the crystal structure of the multidomain HNF4 homodimer with that of the heterodimer RAR-RXR bound to the same DR1con RE, solved by the same authors (45). Here again the same caution applies, since the chosen DNA DR1 sequence is the ideal DR1 sequence which is far from being biologically relevant. RAR/RXR-DR1cons (**Figures 4C, E**) and HNF4/HNF4-DR1cons (**Figures 4D, F**) have a different organization on DR1cons with respect to the positioning of the LBD dimer, which is rotated along the axis of DNA and perpendicularly to it when comparing the two structures. Consequently, the putative conformations of the respective hinge domains must be different, but this information is only partial, since the two hinge regions of RAR/RXR-DR1cons are not resolved in the crystal structure and only the N- and C-termini are modelled as shown in **Figures 4C, E**. Note that topological discrepancies occur (45) between the RAR/RXR-DR1cons crystal structure and the low-resolution solution structure of RARΔAB–RXRΔAB-DR1 (46), with a radically different polarity of the RAR/RXR LBD heterodimer. This is likely due to the fact that the DR1 RE chosen for the solution structure of RARΔAB–RXRΔAB is a natural binding site of DR1 type and not a consensus DR1 RE as used for the crystal structure.

The comparative analysis of the structure of the multidomain HNF4 homodimer on DR1cons with that of the HNF4 DBD homodimer on 3cbb-seq DR1 and with that of RAR/RXR on DR1cons highlights two important issues that should be considered with care when analyzing not only the HNF4 structures, but also structures of nuclear receptors in general. First, the DNA sequence has to be a natural binding site that reflects the biology of the receptor. Second, the lack of critical structural information for the hinge regions due to disorder in a large part suggests that the LBD part still has some flexibility and their conformations will certainly be rigidified upon interactions with coregulators, as suggested in the structures of the transcriptional complexes associated to the estrogen receptor ERα and the androgen receptor AR (47–49). In addition, coregulator/nuclear receptor interactions are synergistically regulated by ligand and DNA (29), as was illustrated in the case of PPARγ-RXRα, where the recruitment of the SRC-2 coactivator to the heterodimer was enhanced through an allosteric communication pathway integrating signals from ligand and DNA (50). Another example of allosteric regulation is the asymmetric recruitment of a single PGC-1α molecule on one of the subunits of the ERRα/γ homodimer (51).

### Localization and structural consequences of mutations involved in MODY1 and other diseases

**Figure 5:**
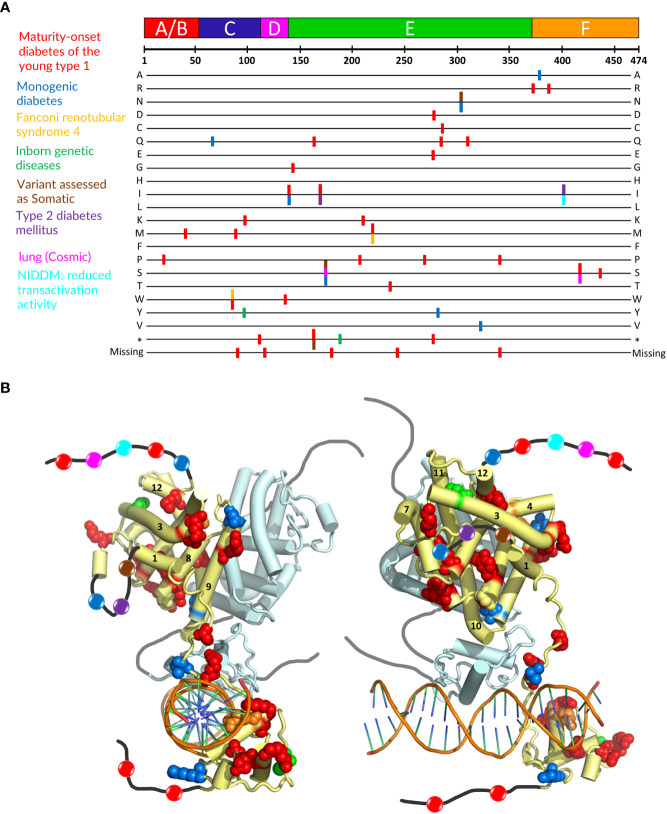
Localization of mutations involved in MODY1 and other diseases. **(A)** Position of the HNF4α mutations that are linked to established diseases. The 474 amino acid sequence of HNF4α is shown at the top together with the receptor domain representation. The next lines correspond to each of the 20 amino acid residues resulting from a mutation event. Note that this also includes the stop codon occurrence and the residue deletion. The colors of the bars correspond to the diseases indicated on the left. **(B)** Sphere representation of the HNF4α mutations shown in **(A)** plotted onto the multidomain HNF4α homodimer structure bound to DR1 RE (PDB: 4IQR). The color code is the same as the one used in **(A)**. If a mutation is involved in several diseases, the mutated residue is shown with the color of the disease that is less frequently encountered. Note that the regions that are not present in the structure (either not considered in the construct, i.e. A/B and F domains, or not modelled, i.e. the hinge regions) are shown as black (dark grey for the second monomer) lines onto which the positions of the mutations are represented as simple colored spheres, with the color according to the affected pathology. Note that one subunit of the HNF4α homodimer is considered for the plotting of the residues, the other one is shown in pale cyan with a tube representation.

**Figure 6:**
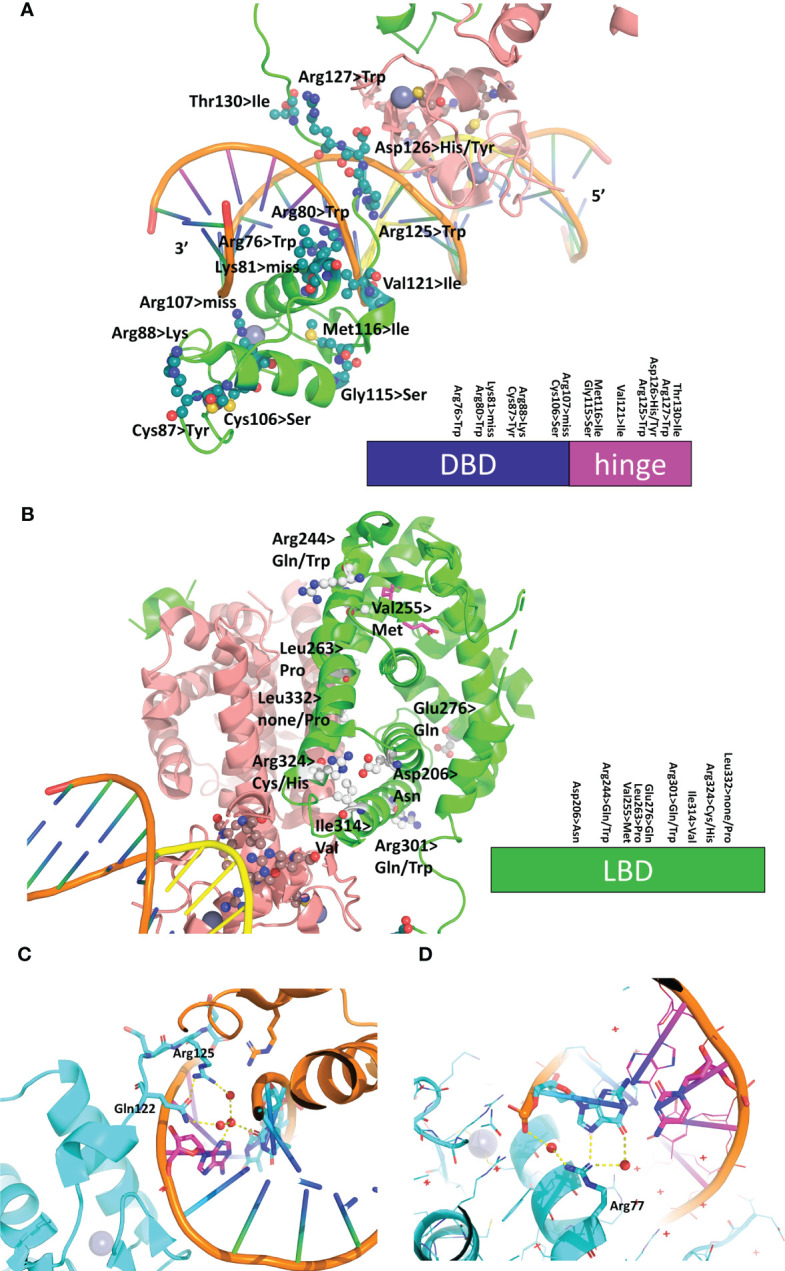
Structural analysis of mutations linked to diseases in the receptor and its RE. **(A)** Mapping of the residues that are mutated in diseases on the DBD-hinge region of the multidomain HNF4α homodimer bound to DR1 RE (PDB: 4IQR). The residues are shown in an all-atom sphere representation and a visual summary along the DBD and hinge region is depicted at the bottom. **(B)** Mapping of the residues that are mutated in diseases on the LBD region of the multidomain HNF4α homodimer bound to DR1 RE (PDB: 4IQR). The residues are shown in an all-atom sphere representation and a visual summary along the LBD is depicted at the bottom. **(C, D)** Structural consequence of mutating base pair in the RE of HNF4-responsive gene promoters, shown for **(C)** the promoter of the factor VII gene where the nucleotide A at position +1 is mutated to C in the disease state and **(D)** the promoter of the factor IX gene where the nucleotide G at position +5 is mutated to C is found in the disease state.

Mutations in the *HNF4α* gene have been found in families with maturity-onset diabetes of the young (MODY), an autosomal dominant form of diabetes characterized by early age at onset and a defect in glucose-stimulated insulin secretion, in families with macrosomia and hyperinsulinaemic hypoglycaemia and in patients with Fanconi renotubular syndrome (52–58)(see https://www.uniprot.org/uniprotkb/P41235/entry for further details). The mutations are found throughout the protein, with a larger number in the DBD and the hinge region and a rather limited number in the N-terminal A/B domain. A visual summary of the distribution of identified mutations thoroughly linked to established diseases is shown in **Figure 5A** according to the type of diseases and their localization is mapped onto the crystal structure of the multidomain HNF4 homodimer bound to DR1cons as shown in **Figure 5B**. As shown in **Figure 6A**, residues that are mutated in the DBD are either found in the DBD zinc finger regions (Cys87 (Cys96) and Cys106 (Cys115)), in direct contact with the half-site (Arg76 (Arg85) and Arg80 (Arg89)) or the half-site spacer (Arg125 (Arg134)), in the hinge regions or participate in the formation of a cooperative homodimer (Asp126 (Asp135)). In the crystal structure of the HNF4α DBD bound to 3cbb-seq DNA, the latter residue Asp126 (Asp135) was thought to make electrostatic interactions with the Arg88 (Arg97) residue of the 5’ DBD subunit. In the structure of the multidomain HNF4 homodimer on DR1cons, the Asp126 (Asp135) residue is oriented in a direction opposite to Arg88 (Arg97) and is close to Arg104 (Arg113) of the 5’ DBD, but does not interact directly with it, it would maybe do so if the receptor would be bound to a more appropriate, natural DNA RE. Mutations in the LBD part are found in the periphery of the receptor core domain (**Figure 6B**); none being involved in direct contact with the ligand. The latter mutations are probably detrimental for the function of HNF4, and cannot be tolerated in agreement with embryonic lethality observed when HNF4 is disrupted in embryos (7). If considering the 3’ DBD subunit, the mutations are localized in the region close to the 5’ DBD (helices 9 and 10 and loop 9-10), in the dimer interface and in other surface regions that are interactions spot for coregulators. Finally, a few mutations are found in the F domain and will affect the interactions of the receptor with protein partners.

### Mutations in the DNA RE linked to hemophilia

In addition to the SNPs and missense mutations affecting the HNF4 protein itself, nucleotide mutations in the HNF4α-binding sites were also reported that were found to be linked to diseases. In particular, mutations in the HNF4α-binding site of the blood coagulation factor VII and IX genes are linked to blood coagulation deficiencies, such as bleeding diathesis and hemophilia B Leyden (59, 60). In the case of the promoter of the factor VII gene, the wild-type HNF4α-binding site reads CGGGCAA*A*GTTCT and in the mutant found in the disease state, the nucleotide A at position +1 (shown in italics in the sequence) is mutated to C. From the structure of the HNF4α DBD homodimer on 3cbb-seq, we observe that the N3 moiety of A (+1) makes ordered water-mediated interactions with Arg125 (Arg134) and Gln122 (Gln131) which are stabilized thereupon (**Figure 6C**). A cytosine nucleotide cannot recapitulate the same interaction pattern and leads to weaker binding interactions with the 3’-DBD. In addition, the change in the DNA sequence resulting from the single nucleotide mutation will lead to a different DNA shape, as can be shown using the DNA shape prediction software (41) and thus affects the electrostatic potential that the protein senses when binding.

Similarly, the wild-type HNF4α-binding site found in the promoter of the factor IX gene reads GTACCTAAGTA*G*A and a mutation in the nucleotide G at position +5 (shown in italics in the sequence) to C is found in the disease state. From the structure of the HNF4α DBD homodimer on 3cbb-seq, we observe that the N7 and O6 moieties of the complementary G to the C at position (+5) makes direct and ordered water-mediated interactions with Arg77 (Arg86) (**Figure 6D**). Mutating the cytosine for a guanine (and thus a complementary cytosine for the interaction with Arg77 (Arg86)) does not provide the required functional groups for interaction with the arginine side chain and weakens the binding with the 3’-DBD. Altogether, these two examples illustrate the fine-tuning effects that take place in the binding of the receptor to its cognate DNA sequences and shows that even a single nucleotide mutation can lead to pathological conditions, emphasizing the allosteric role played by the DNA in the process.

### Structural consequences of functionally important posttranslational modifications

**Figure 7:**
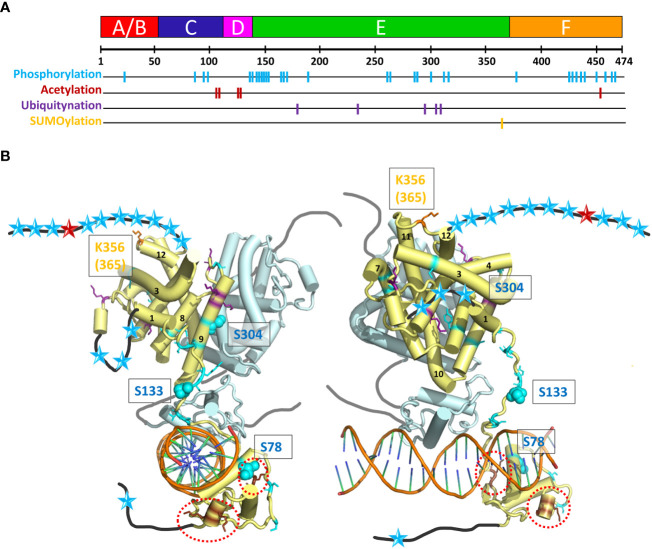
Post-translational modifications (PTM) of HNF4α. **(A)** The 474 amino acid sequence of HNF4α is shown at the top together with the receptor domain representation and the position of the residues subjected to the different types of PTMs are shown with colored bars. **(B)** Sphere representation of the HNF4α PTM shown in **(A)** plotted onto the multidomain HNF4α homodimer structure bound to DR1 RE (PDB: 4IQR). Light blue spheres indicate residues subjected to phosphorylation, brown spheres to residues subjected to acetylation (and are surrounded by red dotted lines, as the residues are difficult to see), purple spheres to residues subjected to ubiquitination and orange spheres to residues subjected to SUMOylation. Note that the regions that are not present in the structure (either not considered in the construct, i.e. A/B and F domains, or not modelled, i.e. the hinge regions) are shown as black (dark grey for the second monomer) lines onto which the positions of the mutations are represented as simple colored stars, with color according to the type of PTM.

HNF4 transcriptional regulatory activities are also controlled by a series of post-translational modifications (PTMs), including phosphorylation, acetylation, ubiquitination and SUMOylation by appropriate enzymes, such as Protein Arginine Methyltransferase 1 (PRMT1), SRC Proto-Oncogene, Non-Receptor Tyrosine Kinase (SRC), Protein Kinase C (PKC), Protein Kinase A (PKA), and AMP-activated protein kinase (AMPK) (61, 62). These different enzymes act upon HNF4α to modify its stability, localization, and DNA binding capacity. A visual summary of the distribution of residues that are subject to PTMs is shown in **Figure 7A** and their localization is mapped onto the crystal structure of the multidomain HNF4 homodimer bound to DR1cons, as shown in **Figure 7B**. Among the different types of PTMs, phosphorylation is the most frequent one, with a particularly high distribution in the hinge, LBD and F domains and a few in the DBD and A/B domains.

### Phosphorylation

The phosphorylation of Ser and Thr residues is one of the most common post-translational modifications (see PhosphoSitePlus https://www.phosphosite.org/homeAction.action). AMP-activated protein kinase (AMPK) has been shown to be a major repressor of the transcriptional activity of HNF4. The phosphorylation of three conserved serine residues can be highlighted in HNF4α. The phosphorylation of Ser78 (Ser87), a highly conserved residue of the DBD has been shown to affect DNA binding, transactivation ability, and protein stability. It also impairs nuclear localization. The long-range action of the phosphoryl group and its proximity to several positively charged and conserved residues (Arg or Lys), which are involved in nuclear localization of HNF4, can clearly affect the DNA/DBD protein interactions. A closer position would most probably totally prevent the binding. Note that phospho-mimetic mutations of the same Ser/Thr residue result in cytoplasmic localization of RXR and PPAR receptors, suggesting that phosphorylation of this conserved Ser residue may be a common mechanism for regulating the function of NRs (63). The functional correlation is less obvious for Ser133 (Ser142) in the hinge domain, only visible in one subunit of the dimer. On this subunit, the position is clearly accessible to contacts with other protein partners (coregulators) and the phosphorylation would then affect the interactions. The AMPK-mediated phosphorylation of HNF4 on serine 304 (Ser313) (64) has a 2-fold effect, reducing the ability of the transcription factor to bind DNA and increasing its degradation rate *in vivo*. The residue Ser304 (Ser313) sits on helix H9 and binds to Glu327 (Glu336) on helix10 of the partner LBD. Its phosphorylation would destabilize the homodimer which explains the two effects.

### Acetylation and ubiquitination

Acetylation of HNF4α was shown to be altered in human decompensated hepatocytes from nonalcoholic steatohepatitis (NASH) and alcohol-mediated Laennec’s cirrhotic explanted livers (65). HNF4α acetylation, a posttranslational modification important for nuclear retention, was significantly reduced in failing human hepatocytes. The alterations in the cMET-AKT pathway directly correlate with the HNF4α localization and the level of hepatocyte dysfunction (66). A comprehensive survey of PTMs in HNF4α protein by mass spectrometry allowed to identify new ubiquitination and acetylation sites (67, 68). To assess their impact in HNF4α function, point mutations at these sites were tested for transcriptional activity. Acetylation at Lys458 in the F domain had a significant impact in the HNF4α-mediated transcriptional control. An acetylation negative mutant at Lys458 showed an increased transcriptional activity by about 2-fold.

### SUMOylation

Like ubiquitination, SUMOylation results in the formation of an isopeptide bond between the c-terminal Gly residue of the modifier protein and the ϵ-amino group of a Lys residue in the acceptor protein (69). SUMOylation plays an important role during hepatocellular differentiation. This is mediated, in part, through regulation of the stability of HNF4α in a ubiquitin-dependent manner. In the SUMO consensus motif Y-K-x-D/E at residues 364–367, the residues Lys365 and Asp367 were mutated to an Arg and an Ala residue, respectively. The use of deletion constructs and point mutants, Lys365 to Arg and Asp367 to Ala demonstrates that the C-terminus in HNF4α is absolutely required for SUMOylation *in vitro*. SUMOylation of HNF4α activation domain 2 (in LBD) may directly affect the conformation of LBD and increase HNF4α transcriptional activity through co-factor binding (70).

The PTMs located at specific residues of the HNF4 homodimer interface affect the receptor dimerization capacity and thus may be lethal for the receptor function (**Figure 7B**). Phosphorylation affects the association to DNA (for example for the residue Ser78), but the position of the majority of the numerous mutations suggests a disruptive role in the binding of various cofactors. Acetylation mutants are concentrated in the DBD and hinge domains. The flexibility of the hinge domain, which is not visible in the subunit shown in light blue in **Figure 7B** suggests that the biologically relevant conformation which prevails in the cell is still different from the ones observed in the crystal structure (where crystal packing effects in large part stabilize the conformation of the hinge region in the 3’-HNF4 subunit). The acetylation of residues in the F domain and the SUMOylation of residues located at the C-terminal end of the LBD strongly support the functional importance of the F domain.

## Conclusions and perspectives

The nuclear receptor HNF4 is a key regulator in the expression of genes involved in metabolic pathways that are prevalent in cell types such as hepatocytes, enterocytes, and pancreatic β-cells. Dysregulation of HNF4 functions is linked to several diseases including diabetes, atherosclerosis, hemophilia and cancer. The structural work carried out over the last twenty years on HNF4 has uncovered key regulatory mechanisms of action of this receptor in terms of ligand binding and DNA binding site selectivity. The resolution of the crystal structures of the HNF4 LBD has opened avenues for pharmacological modulation of the HNF4α activity and the treatment of diseases involving HNF4α, such as type 1 diabetes (MODY1) and cancer (10, 27). Similarly, unravelling DNA binding site preference of HNF4α provides keys for a better understanding of its transcriptional regulatory mechanisms, as illustrated in the comprehensive study of the role of HNF4 in brush border gene regulation (37). Nevertheless, key issues still remain to be answered that have not yet be uncovered by the structural work on the multidomain HNF4 bound to DNA. It certainly provides a glimpse of how the full-length HNF4 receptor would work in the cell, but lacks functionally relevant key aspects. First, structural and functional studies of NRs in general have largely demonstrated the importance of the DNA sequence in the NR functions, in particular in the binding of coregulators as exemplified in the case of the androgen receptor (71). In fact, since DNA is the allosteric effector conveying the action of the NRs, the choice of a biologically relevant DNA sequence is of utmost importance for subsequent structure-function studies. The crystal structure of the multidomain HNF4 was solved bound to an idealized, thus unnatural, consensus DR1 response element. Therefore, atomistic details of the DNA intrinsic shape (resulting from the sequence and from protein-DNA interactions) and of the interactions with the receptor cannot be interpolated to the case of the HNF4 specific binding sites of the type H4-SBM. The hinges of the HNF4 homodimer are crucial for the function of the receptor and the sites of numerous mutations involved in MODY1 and other diseases. In the multidomain structure the hinges are only partly resolved. The hinge of the 5’ subunit is not visible in the electron density and that of the 3’, albeit almost fully built in a poor electron density map, is not predictive of the *in vivo* conformation. In fact, the hinges are interaction spots for transcriptional coregulators and the latter would lock the receptor in a functionally active conformation. Only short coregulator peptides were considered in the structural work. Similarly, no information of the A/B and F domains can be gathered from this study that focused on a ΔA/B plus ΔF HNF4 deletion constructs. To improve our knowledge of the molecular mechanisms of action of HNF4 when bound to its DNA targets and in association with coregulators, structural approaches should integrate cryo electron microscopy studies of functionally relevant endogenous complexes. The correlation with the *in vivo* situation is the next challenge of structural biology. It will be possible to reach this goal in the near future thanks to the important development of cryo electron tomography (72–75). This will lead to a better understanding of the complexity of the biology of HNF4α for designing new therapeutic strategies in the treatment of diseases involving this nuclear receptor.

## Author contributions

All authors were responsible for conceiving, drafting, and critically revising this work, were accountable for the accuracy and integrity of the work, and gave final approval for publishing. All authors contributed to the article and approved the submitted version.

## Footnotes

1 Note that in the following, unless specified, HNF4 will stand for HNF4α
